# Pain as a risk factor for substance use: a qualitative study of people who use drugs in British Columbia, Canada

**DOI:** 10.1186/s12954-018-0241-y

**Published:** 2018-07-05

**Authors:** Pauline Voon, Alissa M. Greer, Ashraf Amlani, Cheri Newman, Charlene Burmeister, Jane A. Buxton

**Affiliations:** 1 British Columbia Centre on Substance Use, 400 - 1045 Howe Street, Vancouver, BC V6Z 2A9 Canada; 2 School of Population and Public Health, Faculty of Medicine, University of British Columbia, 2206 East Mall, Vancouver, BC V6Z 1Z3 Canada; 3 British Columbia Centre for Disease Control, 655 West 12th Avenue, Vancouver, BC V5Z 4R4 Canada

**Keywords:** Pain, Self-management, Harm reduction, Patient-centered care, Risk environment, Methadone

## Abstract

**Background:**

People who use drugs have a significantly higher prevalence of chronic non-cancer pain compared to the general population, yet little is known about how various policy, economic, physical, and social environments may serve as risk or protective factors in the context of concurrent pain and substance use. Therefore, this study sought to explore perspectives, risks, and harms associated with pain among people who use drugs.

**Methods:**

Thirteen focus group interviews were held across British Columbia, Canada, from July to September 2015. In total, 83 people who had lived experience with substance use participated in the study. Using an interpretive description approach, themes were conceptualized according to the Rhodes’ Risk Environment and patient-centered care frameworks.

**Results:**

Participants described how their experiences with inadequately managed pain in various policy, economic, physical, and social environments reinforced marginalization, such as restrictive policies, economic vulnerability, lack of access to socio-physical support systems, stigma from health professionals, and denial of pain medication leading to risky self-medication. Principles of patient-centered care were often not upheld, from a lack of recognition of patients as experts in understanding their unique pain needs and experiences, to an absence of shared power and decision-making, which often resulted in distrust of the patient-provider relationship.

**Conclusions:**

Various risk environments and non-patient-centered interactions may contribute to an array of health and social harms in the context of inadequately managed pain among people who use drugs.

## Background

Pain presents a significant public health concern that is estimated to cost at least $560–635 billion USD annually due to lost productivity and health care costs [1]. The prevalence of chronic non-cancer pain appears to be significantly higher among people who use drugs (PWUD) (48–60%) compared to the general population (11–19%) [2], which may be attributed to inadequate pain management from clinicians who may be hesitant to prescribe pain medications due to potential risks for dependence, misuse, diversion, morbidity, or mortality [3–5]. The escalating crisis of opioid-related overdose across North America has highlighted the extent to which such risks may be manifested. Given the current opioid crisis and the potential role of past prescribing practices on contributing to iatrogenic opioid use disorder [6], recent guidelines have recommended that tighter restrictions be placed on opioid prescribing [7].

Little is known about the experiences of PWUD who seek pain management, and how various policy, economic, physical, and social environments may serve as risk or protective factors in the context of concurrent pain and substance use. A small body of qualitative studies has been published in this area, which have primarily focused on the patient-provider relationship and issues related to stigma, distrust, suspicion, or inexperience in the context of concurrent pain and substance use, which these studies suggest may contribute to compromised therapeutic relationships between PWUD and health care providers [4, 8, 9]. However, the experiences of pain among PWUD have not yet been described in the context of patient-centered care, which has been identified as an important model of care that is associated with improved health outcomes, quality, and safety [10], as well as Rhodes’ Risk Environment, which suggests that the health of PWUD is not simply defined by individual-level factors, but by the complex interplay between policy, economic, physical, and social environments that may serve as risk factors for—or protective factors against—drug-related harms [11, 12]. Therefore, this study sought to explore perspectives on pain management among PWUD in several urban and rural settings across British Columbia, Canada, using the patient-centered care and Rhodes’ Risk Environment frameworks.

## Methods

Focus group data were derived from the Peer Engagement and Evaluation Project (PEEP), a participatory qualitative evaluation of harm reduction services (including opioid agonist and substitution treatment) in British Columbia, Canada. This study has previously been described elsewhere [13–15]. In brief, from July to September 2015, 13 focus groups were held at harm reduction sites across 12 urban and rural locations throughout each of the five regional health authorities in British Columbia. Through local PWUD and service providers, snowball sampling was employed to recruit a total of 83 PWUD. Participants were eligible for inclusion in the study if they self-identified as a person who uses drugs (i.e., at least once in the past week), were 19 years of age or older, had been living in British Columbia in the past 6 months, able to provide informed consent, and able speak and understand English.

Each focus group was facilitated by trained “peer” research assistants (PRAs). Peers were defined as PWUD who draw on their lived experience of substance use to inform their professional work [16, 17]. A PRA-informed question guide sought to elucidate perspectives on barriers and enablers to accessing harm reduction services. All focus group participants provided informed consent and received food, transportation, and a $20 stipend. This study received ethical approval by the University of British Columbia Research Ethics Board (H15-00126).

Following the completion of each focus group, the PRAs and non-peer researchers debriefed to discuss emerging themes and questions that could be changed or added to explore newly identified perspectives in future focus groups. Audio recordings from the focus groups were transcribed verbatim, and data were organized using NVivo 11 software. After the transcripts were cleaned, the researchers and PRAs coded the transcripts using preliminary themes that were conceptualized through debriefing and discussions with the research team. The coded data was then validated during a member checking session with the PRAs. The resulting four major themes from the project included (1) access to harm reduction, (2) readiness for engagement, (3) peer community and networks, and (4) stigma and trust.

The present study describes participants’ experiences of concurrent pain and addiction, which emerged as a recurring topic within the theme of “stigma and trust.” We used the qualitative approach of interpretive description, a non-categorical methodology that seeks to describe themes related to clinical phenomena in order to inform clinical understanding for applied health disciplines [18, 19]. The conceptual frameworks informing this analysis include Rhodes’ Risk Environment and patient-centered care. The Rhodes’ Risk Environment framework suggests that the health of PWUD is not simply defined by individual-level factors, but by the complex interplay between policy, economic, physical, and social environments that may serve as risk factors for—or protective factors against—drug-related harms [11, 12]. The patient-centered care model posits that quality health care must shift away from paternalistic, one-size-fits-all approaches to clinical care, moving instead toward patient-centered approaches that take into account individual differences and preferences related to biopsychosocial factors, subjective health needs and experiences, shared power and decision-making, and communication and relationships built upon mutual trust [11].

## Results

A summary of the participants’ socio-demographic and drug use characteristics is shown in Table 1. As summarized below and in Table 2, several themes related to Rhodes’ Risk Environment and patient-centered care emerged in the focus group discussions on pain management. The following excerpts are labeled according to focus group number, gender (e.g., female (F) or male (M)), and participant number.

**Table 1:** Socio-demographic and drug use characteristics of participants in the Peer Engagement and Evaluation Project (PEEP) (*n* = 70^a^)

Characteristic		
Age	Mean (years)	Range (years)
All clients	44	18–64
Female	41	18–60
Male	45	20–64
Gender	*n*	%
Female	30	42.9
Male	38	54.3
Transgender	1	1.4
Other	1	1.4
Ethnicity	*n*	%
Aboriginal	25	35.7
Non-Aboriginal	45	64.3
Income source^b^	*n*	%
Full-time employment	0	0
Part-time employment	6	8.6
Self-employed	7	10.0
Disability assistance	42	60.0
Social assistance	20	28.6
Other	13	18.6
Housing status	*n*	%
Owned unit	3	4.3
Rental unit	38	54.3
Shelter	10	14.3
No fixed address	16	22.9
Other	3	4.3
Drugs used in the last week^b^	*n*	%
Heroin	30	42.9
Methadone	19	27.1
Morphine	28	40.0
Dilaudid	15	21.4
Oxycodone	12	17.1
Fentanyl	15	21.4
Benzodiazepine	15	21.4
Cocaine	27	38.6
Crack	37	52.9
Crystal Meth	36	51.4
Stimulant	12	17.1
Marijuana	10	14.3
GHB	2	2.9
Suboxone	3	4.3
Acid (LSD)	3	4.3
Ecstacy	1	1.4
Tylenol #3	1	1.4
Number of drugs used in the last week	*n*	%
0	2	2.9
1	8	11.4
2	7	10.0
3	18	25.7
4	15	21.4
5	7	10.0
6	2	2.9
7	6	8.6
8	1	1.4
9	3	4.3
10	1	1.4
Method of drug use in the last week^b^	*n*	%
Smoke	69	98.6
Snort	61	87.1
Inject	44	62.9
Swallow	54	77.1
Other	8	11.4

^a^Missing responses due to incomplete demographic forms in one rural region

^b^ Individuals were able to provide more than one answer

**Table 2:** Summary of Rhodes’ Risk Environment and patient-centered care themes related to pain management that emerged from 13 focus groups of people who use drugs in British Columbia, Canada, from July to September 2015 (*n* = 83)

Framework	Element	Example
Rhodes’ Risk Environment Suggests that the health of people who use drugs is not simply defined by individual-level factors, but by the complex interplay between policy, economic, physical, and social environments that may serve as risk factors for—or protective factors against—drug-related harms.	Policy environments	Restrictive policies regarding treatment of pain and opioid agonist treatment E.g., “And I talked to him [methadone doctor] about my lower back pain and he looked right at me and goes, ‘No, no, I took you on as a patient but I won’t take you on as a pain patient.’”
	Economic environments	Economic factors contributing to higher-risk drug use E.g., “… doctors up here won’t give pain meds … that’s why some people end up going on the street and doing needles because you spend $40 on a pill, you don’t wanna eat it and not get anything out of it.”
	Physical environments	Geographic differences in access to socio-physical support systems that facilitate access to pain management E.g., “So behind the times… for chronic pain issues and I mean… in Vancouver I could’ve went over to [harm reduction program] and they’d send an advocate with me over to that place… down there you do not have to just go ask another doctor.”
	Social environments	Stigma from health professionals as barriers to pain management; denial of pain medication reinforcing marginalization and risky self-medication E.g., “Once you’re flagged, you’re flagged … they have legitimate pain they’re just gonna go to the street and get something”
Patient-centered care Suggests that quality health care must shift away from paternalistic, one-size-fits-all approaches to clinical care, moving instead toward patient-centered approaches that take into account individual differences and preferences.	Recognition of bio-psychosocial influences on health; acknowledgement of subjective health needs and experiences	Patients as experts in recognizing biopsychosocial differences and subjective health needs and experiences related to pain management E.g., “…everybody’s pain is different right? Some people’s tolerance to medication is they can handle more medication, not that they’re getting stoned or whatever, it just takes more to get rid of that pain.”
	Shared power and decision-making between patients and health care providers; promotion of patient-provider communication and relationships based on mutual trust	Absence of shared power and decision-making in pain treatment plan contributing to distrust of the patient-provider relationship E.g., “Doctors give you false information … let’s say you’ve been on morphine for 10 years for chronic pain issues and then doctors will try and…some doctors will try and get you to go on methadone when methadone does not work for pain, not for a lot of people.”

### Restrictive policies regarding treatment of pain and methadone maintenance treatment

In the study setting, many methadone maintenance treatment (MMT) clinics have strict policies against treating patients for concerns ‘unrelated’ to their methadone treatment, despite the often comorbid nature of pain and opioid use disorder. Several participants were enrolled in MMT and recounted how such restrictive policies served as barriers to effective pain management in their interactions with physicians:

1-M3: And I talked to him [methadone doctor] about my lower back pain and he looked right at me and goes, “No, no, I took you on as a patient but I won’t take you on as a pain patient.”

Other participants described their experiences with similar barriers in acute care settings:

2-M6: If you use methadone and go on a methadone program, then, then you injure yourself and you need painkillers and go to a hospital, and they find out you’re on a methadone program, you don’t get anything more than an aspirin.

### Economic factors contributing to higher-risk drug use

Participants expressed how their inability to obtain pain treatment from physicians led them to obtain diverted or illicitly manufactured pain medication from street-based drug markets, and how economic factors influenced their decisions to use such pain medications via high-risk injection practices (e.g., in order to achieve a more potent or rapid effect, as opposed to consuming pain medications via oral administration):

1-F1: I mean doctors up here won’t give pain meds.

1-F2: They won’t give you anything.

1-F1: Like so I mean that’s why some people end up going on the street and doing needles because you spend $40 on a pill, you don’t wanna eat it and not get anything out of it.

In light of the economically vulnerable position of many PWUD, participants described the protective potential of applying harm reduction principles in the context of pain management among people who actively use drugs:

1-F1: Harm reduction would be the doctors prescribing [to] people who are in pain.

1-F2: Yeah. Yeah. If I had my own prescription, I wouldn’t be selling it, I’d be using it and I wouldn’t be spending all my money on pain killers.

Participants also expressed how the financial strain of obtaining diverted or illicitly manufactured pain medications subjected them to economic vulnerability heightened by competing familial demands:

3-M3: You know I’m paying $30 a pill for my pain meds for actual, you know, for true chronic pain like…[gets cut off].

3-F1: [Inaudible]…and we have two teenaged daughters, how the fuck are we supposed to [pay] 30 bucks a pill?

### Geographic differences in access to socio-physical support systems that facilitate access to pain management

Participants expressed how the culture of practice among physicians varied by geographic location. Resoundingly, participants in rural regions described their fear of being “outed” as a PWUD in their community as their access to pain management would be compromised. In rural towns, participants often had access to only one physician. One participant in a rural location stated, “doctors up here won’t give pain meds,” while others expressed:

4-F2: [Man who was shot] was an addict so they wouldn’t give him anything.

4-M2: That’s something else altogether.

4-F2: Is that not cruel and unusual?

4-F4: Yes it is and it doesn’t happen at all the hospitals because [Name], when he OD’d, he went to [rural town] and he was looked after really well as far…so I mean, I think this particular hospital and the attitude of all these [expletive omitted] doctors suck.

4-M1: We’re all labeled…we’re all labeled.

However, even in urban centers, barriers to accessing medical care for pain management were expressed, such as difficulties walking to public transportation while in pain. Geographic differences were also observed in relation to accessibility to socio-physical support systems for PWUD. For instance, a specialized addiction clinic with staff who could help advocate for PWUD was accessible in an urban city, compared to a smaller rural setting without such supports:

3-M3: So behind the times… for chronic pain issues and I mean… in Vancouver I could’ve went over to [harm reduction program] and they’d send an advocate with me over to that place… down there you do not have to just go ask another doctor.

Similarly, a larger urban setting provided access to approachable staff and a culture of care that may have been fostered in part by a well-established peer advocacy group:

4-F2: […] I got stuck in Vancouver once when I lived in Abbotsford and I have back issues and so I had some severe sciatic pain and called them at [harm reduction program] because I’d been there and talked to people there and they told me how to get on the bus and get to [hospital]. I walked in the door… front door of [hospital] where they have their initial triage, they sent me down to Fast Track and from the minute I walked in the door to the minute the doctor saw me, it was 24 min.

4-F4: That’s an awesome hospital it is.

4-F2: Right, and he […] immediately gave me something for pain and prescribed Percocets for me, which is what I can take because I am allergic to other things and was no problem. Now I know that [peer-based organization] has worked countless hours and years with [hospital] but perhaps we should get some hints from them…those two groups to see about training the hospital staff.

### Stigma from health professionals as barriers to pain management

Participants commonly expressed their experiences of addiction-related stigma from health professionals, which served as a barrier to accessing pain management:

5-F3: Like if you go…me having HIV, I go up to the hospital, some of the nurses up there are really nice but a lot of them are kinda like…just look at you…like you just feel disgusting the way they look at you and if they know you’re an addict, they’ll push…if…if you go to the emergency room and you’re there first, you’ll be the last one seen. They think you’re just there trying to get drugs or…or whatever, it doesn’t matter if you’ve got a broken arm and it’s hang out to your frikken’ elbow, you’re elbow’s popped out, you’re gonna be the last one seen, like it’s that bad up here.

5-M2: And you won’t get any pain killers.

5-F3: No, they’ll maybe give you a couple Advil and send you home.

Other participants expressed how inadequate pain treatment may stem from a perception that such treatments may be “enabling”:

4-F2: So it’s not like you’re abusing it, you know, but they’ve got this idea in your head, their heads that if you’re an addict then you don’t deserve anything for….

4-F4: Treatment, that’s right.

4-F2: You don’t deserve anything for pain and they’re not gonna enable.

### Denial of pain medication reinforcing marginalization and risky self-medication

Many participants expressed their experiences with being unable to obtain pain medication from clinicians, which led them to subsequently self-manage their pain by obtaining diverted or illicitly manufactured pain medications, thereby reinforcing marginalization and entrenchment in street-based drug markets. Examples include “And I said, you know, ‘You don’t give me the pain meds I gotta go get ‘em, I’m in chronic pain’” [1-M3], “I wouldn’t be…have much to do with the street scene if the doctor gave me the pain killers” [1-F2], “It wouldn’t be such a black market if the doctors didn’t punish us for past self-medicating” [1-F2], “Once you’re flagged, you’re flagged and I mean that just…if you…they have legitimate pain they’re just gonna go to the street and get something” [4-F4], and “I didn’t have a doctor and I self-medicated myself because of the pain right? And, uh, lo and behold here I am trying to get an operation. I’ve been like this for a year and a half” [6-M4]. One participant even reflected on the unethical nature of individuals having to resort to managing their pain on their own, stating “That’s what I mean, by the doctors turning people away like that, look what happens … Self-medicating…yeah. That’s inhumane” [4-F4]. For some, the inability to access pain medication contributed to high-risk injection practices:

1-F2: The doctors won’t prescribe him pain medication because he used needles….

1-M3: And I was only using needles because he cut me off and I told that to my doctor and he said, you know, he’s like, “Oh, so you been using needles the whole time,” and I’m like, “No, a year…I started up about a year ago ‘cause you cut me off first.”

### Patients as experts in recognizing biopsychosocial differences and subjective health needs and experiences related to pain management

As experts with insight into their own health needs, participants expressed an understanding of pain treatment concepts such as tolerance, and the person-specific nature of pain treatment:

1-M2: I would like to know where they draw, uh, everybody’s pain is different right? Some people’s tolerance to medication is they can handle more medication, not that they’re getting stoned or whatever, it just takes more to get rid of that pain. […] Well what I’d like to say is that we come out with a scale or… “Okay, this pain regulates that much more pain, or this much Oxycontin or…” whatever right? And, you know, put somebody on that amount but my doctor once said he had somebody 300 lbs doing the same amount as me, couldn’t get off the couch.

1-F2: Well he’s got a lower tolerance.

1-M2: And that…that’s my point like I had to go and buy more … Because he can’t prescribe any more.

### Absence of shared power and decision-making in pain treatment plan contributing to distrust of the patient-provider relationship

The patient-centered care principles of shared power and decision-making, communication, and relationships built upon mutual trust emerged as particularly fractured in participants’ experiences in the context of pain management. Specifically, methadone was seen as a treatment option that lacked shared power and decision-making:

1-F1: They’re trying to put everybody in pain on Methadone and Methadone does not work for everybody but they don’t care […] It’s Methadone or nothing.

1-F2: Yeah, I’ve got back issues but because I’ve self-medicated in the past, I don’t…I’m not allowed to get any prescriptions for back pain or anxiety.

Distrust was also evident in participants’ perceptions of methadone, exemplified by statements such as “Now they’re lying about methadone, ‘Oh, it’s one of the purest, most excellent pain medicines in the world and you won’t get anything better.’ It’s all a lie. It’s a complete lie” [1-M3], and “Doctors give you false information … let’s say you’ve been on morphine for 10 years for chronic pain issues and then doctors will try and…some doctors will try and get you to go on methadone when methadone does not work for pain, not for a lot of people” [3-M3].

Another common theme that appeared to strain patient-provider relationships was being suddenly “cut off” pain prescriptions with little communication or shared decision-making:

2-M1: I don’t know why but everybody’s been getting cut down or cut off on opiates and everybody, chronic pain, like I got a broken neck and I’ve got chronic pain, I’m sure multiple people have the same situation, it’s just chronic, always in pain. Now for a doctor to turn around and say like my doctor said to me, people are selling ‘em on the street, blah, blah, blah, blah, blah, therefore I’m cutting you down to half a dose and then we’re going to wean you off within a 2 week period. Well, does that give me a new neck? No, it didn’t do absolutely nothing.

## Discussion

Collectively, these findings highlight the ways in which various levels of Rhodes’ Risk Environment may reinforce marginalization in the context of inadequately managed pain among PWUD, such as program-level MMT policies, economic vulnerability, lack of access to socio-physical support systems, stigma from health professionals, and denial of pain medication leading to risky self-medication. Furthermore, these findings highlight how the principles of patient-centered care are often not upheld in the context of pain management among PWUD, from a lack of recognition of patients as experts in understanding their unique pain needs, to an absence of shared power and decision-making, which often results in distrust of the patient-provider relationship.

Complexities surrounding the intersection between pain management and MMT emerged as a recurring theme throughout the present study. Despite the high prevalence of chronic pain among individuals on MMT (estimated to be between 55 to 61%) [20, 21], many clinicians are reluctant to prescribe pain medication to MMT patients; may view MMT as a treatment for either pain or addiction separately; or may prescribe MMT as a one-size-fits-all approach to managing concurrent pain and opioid dependence, without taking into account patient-centered care principles such as biopsychosocial differences and individual preferences [22]. For instance, literature has found that significantly higher doses of MMT may be required for individuals with concurrent chronic pain [23]; metabolism of methadone varies considerably between individuals [24]; and that a range pain management options exist for individuals on MMT, such that clinicians should not have to simply deny pain medication altogether [25].

Furthermore, as illustrated in the present findings, stigma and inadequately managed pain appear to be common among MMT patients, with 78% reporting MMT-related stigma in one study [26], and several studies reporting undertreated pain among individuals on MMT [21, 27–29]. As such, it is perhaps not surprising that many PWUD with pain are distrustful of the “methadone or nothing” approach, and often self-manage their pain instead of, or in addition to, methadone treatment [26]. Thus, clinicians may wish to explore alternative approaches for treating pain, particularly in the context of opioid use disorder, such as buprenorphine/naloxone and specialist-led approaches, which have recently become more widely implemented in the study setting since the time of this study’s data collection [30]. Specifically, buprenorphine/naloxone has become the recommended first-line option for pharmacological treatment for opioid use disorder, due to its superior safety profile compared to methadone [30]. Additionally, specialist-led approaches for treating opioid use disorder such as maintenance treatment with slow release oral morphine or injectable opioid agonists (e.g., hydromorphone, diacetylmorphine) have also been implemented in the study setting [30]. Given that little is known about the effect of these non-methadone alternatives on pain management, patient preference, engagement and retention in treatment, and other factors such as perceived stigma among PWUD, these are important areas that warrant exploration in future research.

Self-managing pain among PWUD evidently poses high risk for morbidity and mortality such as injection-related harms (e.g., infection, HIV/HCV transmission) or overdose, especially given recent concerns related to fentanyl contamination in the illicit drug market [31, 32]. In the present study, PWUD described their experiences with self-medicating after being unable to obtain pain medication from health providers. This phenomenon has previously been described in other literature. One study found that 97% of people who inject drugs reported self-managing pain via injecting heroin (52%) or obtaining diverted or illicitly manufactured pain medication from street-based drug markets (65%) [33]. Additionally, 66% of people who inject drugs reported they had been denied pain medication, often for reasons such as perceived drug-seeking (44%), clinic policies restricting narcotic prescribing (26%), or being told that methadone is sufficient for pain (18%), as echoed in the present findings [34]. Furthermore, denial of pain medication significantly predicted in-hospital illicit drug use [35].

These findings also demonstrate a range of psychosocial harms related to inadequately managed pain among PWUD, from economic vulnerability due to the financial strain of buying street-based pain medication, to perceptions of “inhumane” and “cruel and unusual” treatment from health care providers. Such factors may perpetuate and reinforce the marginalization of PWUD, such as economic vulnerability leading to high-risk injection drug use, and perceived stigma leading to avoidance of health care and subsequent risky self-medication. Indeed, other research has found that factors related to economic vulnerability (e.g., drug prices, perceived cost-effectiveness) may determine risky drug use practices, including initiation into injection drug use [36], and that stigma from health professionals in the context of pain among PWUD may lead to further harms (e.g., withdrawal) and compromised therapeutic relationships [8]. To mitigate these harms, strategies are needed to improve pain management for PWUD, such as improving access to peer advocacy groups that may support PWUD and facilitate education, as was endorsed by participants in the present study.

This study has limitations that should be noted. First, it is often difficult to disentangle the complex interplay between physical and emotional pain. Second, while focus groups can have the advantage of encouraging participation and active discussion [37], this methodology also poses inherent areas for potential bias, such as tendencies toward normative discourse [38]. Third, the excerpts presented represent individuals who were identified by the transcriptionist as gender-binary (i.e., male or female voice) due to the inability to connect participants’ self-reported demographics to those speaking within the focus groups, in order to maintain confidentiality. While two participants in the study identified as non gender binary (i.e., transgender or other; see Table 1), the lack of non-binary gender diversity represented in the focus group quotations is a limitation of this study. In general, representation of non-binary gender perspectives in the field of pain and addiction research is limited; thus, this presents an important area for future exploration.

## Conclusions

As the opioid crisis continues to devastate communities of PWUD across North America, these findings shed light on lesser-described phenomena of self-managed in the context of various risk environments, which could be contributing to high-risk opioid use. Recent guidelines on prescribing opioids for chronic pain propose scaling back on opioid prescribing [7]; however, without providing effective analgesic alternatives for individuals who use opioids for pain, these findings (notably gathered before the release of these recent guidelines) illustrate how denial of pain medication may paradoxically put individuals at risk for self-harm, while potentially also damaging trust in the patient-provider relationship and diminishing individuals’ willingness to seek maintenance treatments for opioid use disorder. Therefore, more research is needed to investigate trends in self-management of pain in light of developing policy discussions. For instance, prescription drug monitoring programs (PDMPs) are being widely implemented in many settings; however, the effectiveness of these systems, particularly from the perspective of PWUD with pain, has not been well established [39]. In fact, one recent systematic review found insufficient evidence of effectiveness of PDMPs, and even highlighted three studies that found an increase in heroin overdoses after PDMP implementation [40]. Other major policy discussions in the study setting relate to cannabis legislation, which emerging research suggests may play a role in mitigating the opioid crisis through reduced opioid prescribing [41, 42]. Added to this, the potential role of cannabis in pain management among PWUD will be an important area for future research.

Certainly, the potential for fatal overdose and other severe harms that may be associated with opioid analgesics is not to be understated, yet equally important is the “need to engage patients in an honest and open way rather than quickly writing or refusing to write opioid prescriptions” [43] or consider other pain management approaches (e.g., non-opioid pharmacological, interventional, psychological, physical rehabilitation, or complementary and alternative approaches) in ways that take into account individuals’ complex risk environments and patient-centered care principles.

## Abbreviations

MMT: Methadone maintenance treatment
PDMP: Prescription drug monitoring program
PEEP: Peer Engagement and Evaluation Project
PRA: Peer research assistant
PWUD: People who use drugs
